# Risk Factors for Diabetic Retinopathy among Jordanian Diabetics

**DOI:** 10.4103/0974-9233.51997

**Published:** 2008

**Authors:** Muawyah D. Al-Bdour, Maha I. Al-Till, Khawla M. Abu Samra

**Affiliations:** From the Department of Ophthalmology, Jordan University Hospital, Amman, Jordan

**Keywords:** diabetes mellitus, diabetic retinopathy, Jordan, risk factors

## Abstract

**Purpose:**

To identify the risk factors associated with diabetic retinopathy among diabetic patients at Jordan University Hospital.

**Methods:**

A total of 986 patients with diabetes mellitus were assessed at Jordan University Hospital. The assessment included detailed relevant history, complete medical and ophthalmic evaluation.

**Results:**

Out of the 1961 eye examined, 64.1 percent had one form of diabetic retinopathy; 54.8 percent had nonproliferative diabetic retinopathy (NPDR), 9.3 percent had proliferative diabetic retinopathy (PDR) and 30.8 percent had maculopathy. Of all participants, 23.5 percent had combined NPDR and maculopathy while 7.6 percent had PDR and maculopathy. Using logistic regression analysis it was shown that old age group, long duration of diabetes, poor glycemic control, uncontrolled blood pressure and the presence of nephropathy were significantly associated with diabetic retinopathy. The incidence of maculopathy was significantly associated with the presence of hypertension, protienuria and high cholesterol level.

**Conclusion:**

The challenge for the primary care physician and diabetologist is to attain excellent glycemic control, aggressive control of blood pressure and normalization of blood lipid in order to reduce the risk of blindness and lessen the burden from diabetic retinopathy.

Diabetes mellitus is a complex multifactorial disease, often associated with progressive visual loss. Diabetic retinopathy is the leading cause of new cases of blindness in person aged 20-74 years in the western world.^1^–^3^ The prevalence of blindness and visual impairment among Jordanian diabetics were found to be high.^4^

In the last few decades many large clinical trials have shown that timely controlled photocoagulation reduces visual loss and blindness from diabetic retinopathy.^5^,^6^ These findings highlight the importance of early detection and treatment.

The risk of retinopathy is directly related to the control and duration of diabetes as shown by the Wisconsin Epidemiology Study of Diabetic Retinopathy (WESDR).^7^,^8^ Although other risk factors are also known, their objective influence on the development of diabetic retinopathy are not well studied. The identification of risk factors associated with diabetic retinopathy is essential if preventive measures are to be adopted and it is important for the development of better management strategies for diabetic retinopathy.

This study aims to identify risk factors for diabetic retinopathy in a diabetic population receiving treatment at Jordan University Hospital in order to facilitate the planning of screening service in Jordan.

## Method

In this cross sectional study, 986 patients with diabetes mellitus were assessed at the ophthalmology department/ Jordan University Hospital in Amman.

The ophthalmologic evaluation was conducted by one of two consultants and included: best corrected visual acuity (BCVA) using Snellen chart, pupillary reflexes, detailed slit lamp examination, tonometry using Goldmann's applanation tonometer and dilated fundus exam by indirect biomicroscopy.

After signing a formed consent all the 986 patients included in the study underwent a complete medical assessment. The assessment was conducted by specialist physician and a well-trained staff.

The evaluation included blood pressure and body mass index measurement. The patients also underwent a range of laboratory tests which included; fasting blood sugar, HbAIC, lipid profile, kidney function test and urine analysis. Protienuria was looked for and determined using commercial urine strips.

Blood pressure was measured using a conventional mercury sphygmomanometer. Hypertension was deemed to be present when the recorded blood pressure was higher than 140/90 in at least two occasions.

Retinopathy was classified according to the modified Airlie House classification, as introduced by the Early Treatment Diabetic Retinopathy Study (ETDRS).^9^ In which DR is classified into nonproliferative (NPDR), prolifrative (PDR), and maculopathy. NPDR was further subdivided into mild (microaneurysms confined mainly to the area temporal to the fovea), moderate (vascular changes seen in one to two quadrants of the retina) and severe (vascular changes seen in more than two quadrants). PDR was classified into neovessls at the disc(NVD), neovessls elsewhereb(NVE) and advanced PDR.

Data analysis was carried out using the statistical package for social sciences (SPSS). The independent variables used in the logistic regression analysis were age, gender, duration of diabetes, diabetes control, presence of hypertension, lipid profile, presence of nephropathy and protienuria.

## Results

At the beginning of this study one thousand patients were initially evaluated. Of these, 4 patients with gestational diabetes and 10 patients with diabetes duration of less than 2 years were excluded from the study.

Of the 986 patients enrolled in the study, the age ranged between 9and 86 years with a mean age of 55.3 years (SD 12.5). Most of the patients (50.1 percent) were in the age group 40-59 years (Table 1). Males outnumbered females in the study group being 53.2 percent.

**Table 1:** Age Distribution of the Study Population

Age Group (years)	Number	%
9-19	20	2.0

20-39	63	6.4

40-59	494	50.1

60	409	41.5

Total	986	100

In our study, 6.7 percent of patients were of type 1 diabetes and 93.3 percent were of type 2.Out of the 1961 eye examined, 64.1 percent had one form of diabetic retinopathy;54.8 percent had NPDR, 9.3 percent had PDR and 30.8 percent had maculopathy. Of all participants, 23.5 percent had combined NPDR and maculopathy while 7.6 percent had PDR and maculopathy (Table 2).

**Table 2:** Retinopathy among the Study Population (No of Eyes = 1961)

Variable	Number	%
NPDR	1075	54.8

Mild	466	23.7

Moderate	290	14.8

Severe	319	16.3

PDR	183	9.3

NVP*	41	2.1

NVE**	53	2.7

Advanced	89	4.5

Maculopathy	604	30.8

Exudative	260	13.2

Ischemic	115	5.9

Mixed	70	3.6

Exuda.+ CSME°	121	6.2

Mixed + CSME	38	1.9

* Neovessels at the disc

** neovesels elsewhere

° Clinically significant macular edema.

Subjects with type 2 diabetes had a significantly higher prevalence of both NPDR and maculopathy (P<0.001). On the contrary, the prevalence of PDR was significantly higher in type 1 than type 2 (P=0.007)

In this study, the mean duration of diabetes was found to be 11.9 year with most patients (48.8 percent) in the range 10-19 years. It was obvious that patients with retinopathy significantly had a longer mean duration of diabetes and that the prevalance of total retinopathy increased significantly with increasing duration of diabetes Table 3.

**Table 3:** Diabetic Retinopathy and Duration of Diabetes

Duration (years)	NPDR (%)	PDR (%)	Maculopathy (%)
3-9	39.2	2.4	13.9

10-19	63.8	10.1	38.0

20– 29	66.7	25.0	50.0

30	62.9	31.4	68.6

P-value	<0.001	<0.001	<0.001

There was a significant association between the prevalence of retinopathy and the control of diabetes. Subjects with poor glycemic control as indicated by a high glycosylated haemoglobin values had a higher prevalence of retinopathy than those with a good control of their diabetes as shown in Table 4.

**Table 4:** Diabetic Retinopathy and HBA1C

HbA1C	NPDR (%)	PDR (%)	Maculopathy (%)
< 7.0	47.7	6.8	19.2

> 7.0	57.5	11.3	37.0

P-Value	0.003	0.022	<0.001

Hypertension was detected in 59.8 percent of the patients, 95.7 percent out of these were on medical treatment.Our study showed that NPDR and maculoapthybut not PDR, were significantly associated with high blood pressure Table 5.

**Table 5:** Diabetic Retinopathy and Hypertension

BP	NPDR (%)	PDR (%)	Maculopathy (%)
High	57.5	11.2	34.5

Normal	48.5	7.5	2.5

P-Value	0.005	0.058	<0.001

Examination of urine showed that 70 percent of patients had positive values for protienuria, which was significantly related to PDR and maculopathy, but not to NPDR as shown in Table 6.

**Table 6:** Diabetic Retinopathy and Protienuria

Protienuria	NPDR (%)	PDR (%)	Maculopathy (%)
Positive	57.8	13.5	40.1

Negative	53.0	6.4	24.0

P- Value	0.225	0.002	<0.001

The study showed a significant association between the development of diabetic maculopathy and hypercholesteremia, but not to the development of PDR and NPDR (Table 7).

**Table 7:** Diabetic Retinopathy and Hypercholesterolemia

Serum Cholesterol mmol/l	NPDR (%)	PDR (%)	Maculopathy (%)
5.7	52.9	9.0	28.2

>5.7	58.1	11.1	35.5

P-Value	0.192	0.364	0.044

## Discussion

In Jordan, DM is a common disease with overall prevalence of 13.4% over the age of 25 years.^10^

In a hospital-based study, DR was shown to be the leading cause of blindness above the age of 20 years.^11^ In another study the prevalence of blindness and visual impairment among Jordanian diabetics were found to be 7.4 percent and 10.3 percent, respectively.^4^

The aim of our cross sectional study was to investigate the prevalence and risk factors of diabetic retinopathy in a group of diabetics though we believe that the patients included in the study are not representative of all patients with diabetes in Jordan, but rather a selected group with more advanced disease.

The prevalence of diabetic retinopathy among our patients was found to be 64 percent, which is higher than that found in other studies from Australia, Denmark, Iceland, Sweden, United States and United Kingdom which ranged at 24-62 percent.^12^–^19^ In our region, the prevalence of DR seems also to be high. It was 31.3 percent in Saudia Arabia and 42.4 percent in Oman.^20^,^21^

Our study showed that the duration and the prevalence of diabetes were closely related, confirming results from previous studies.^17^,^18^,^22^ The longer the duration of diabetes the higher the prevalence of total retinopathy and the higher the prevalence of NPDR, PDR and maculopathy. After a duration of 30 years of diabetes the prevalence of NPDR, PDR and maculopathy were 62.9, 31.4 and 68.6 percent respectively. These data stress the need for periodic ophthalmic evaluation for all diabetic patients.

In the current study, poor control of diabetes as indicated by glycosylated haemoglobin levels was a significant risk factor for retinopathy. Patients with a good diabetic control had a lower prevalence of diabetic retinopathy than those with poor glycemic control. Several studies showed an obvious relationship between glycosylated haemoglobin and the incidence and progression of diabetic retinopathy.^23^–^25^

Numerous previous studies supported the association between hypertension and the development and progression of retinopathy.^7^,^8^,^26^ Our study showed a strong relation between hypertension and the prevalence of diabetic retinopathy, in which high blood pressure was significantly associated with NPDR and maculopathy. No significant association was found between hypertension and PDR. The WESDR showed a significant association between the presence of macular oedema and high blood pressure, which is consistent with our findings. These data suggest that better control of blood pressure in diabetic patient may be a useful adjunctive therapy to prevent the progression of retinopathy.

Our study showed a positive relation between diabetic retinopathy and hypercholesterolemia in which it was significantly associated with the development of maculopathy, but not NPDR or PDR. Similarly the WESDR found a significant association between diabetic retinopathy and cholesterol level in insulin treated diabetic patients.^27^

Nephropathy, which assumed to result from microvascular complications of diabetes, was found to be significantly associated with PDR and maculopathy but not NPDR. In the WESDR there was a strong relation between nephropathy, as indicated by the presence of protienuria, and the severity of retinopathy in all age groups.^7^,^8^ Recently the WESDR found that gross proteinuria increased the risk of development of PDR in younger onset patients with diabetes.^28^

We conclude that increasing duration of diabetes, increasing age, poor glycemic control, presence of nephropathy, hypertension and high cholesterol level are significantly associated with diabetic retinopathy. These data support the need for controlling blood sugar, blood pressure and blood cholesterol to reduce the risk of diabetic retinopathy.
